# Diverse effects of calcineurin in vascular smooth muscle cells: physiological activators and the controversial actions of clinical inhibitors

**DOI:** 10.3389/fphar.2026.1819168

**Published:** 2026-04-24

**Authors:** Alexander Nolze, Claudia Grossmann

**Affiliations:** Julius Bernstein Institute of Physiology, Martin Luther University Halle-Wittenberg, Halle, Germany

**Keywords:** calcineurin, cyclosporine A, hypertension, tacrolimus, vascular smooth muscle cells

## Abstract

Calcineurin is a serine/threonine phosphatase that classically regulates T cell activation and modulates immune response by targeting transcription factors of the NFaT family. Activation of calcineurin for example by angiotensin II, phenylephrine, endothelin-1 or mechanical stress can influence vascular smooth muscle cell function and stimulates proliferation and migration or affect the phenotype of these respective cells. This can lead to vessel wall remodeling, increased vascular tone or fibrosis, which contribute to the development of cardiovascular diseases. Based on its classical function, inhibition of calcineurin activity by calcineurin inhibitors is a common treatment in the clinics for autoimmune and inflammatory disease or to prevent graft rejection. Classical calcineurin inhibitors can promote pathological effects in vasculature that resemble calcineurin activation, namely the development of systemic hypertension or inflammatory processes, making the interpretation of the role of calcineurin in vascular smooth muscle cells difficult. In this mini review, we provide a summary of known pathological outcomes of calcineurin activation and calcineurin inhibitor-induced effects in vascular smooth muscle cells. Knowledge about these functional alterations can provide a useful tool to avoid negative effects for the vasculature during pharmacological intervention. Overall, maintenance of a balanced calcineurin activity is essential for proper vascular smooth muscle cell function.

## 1 Introduction

Calcineurin (PPP3C) is a calcium- and calmodulin-regulated serine threonine phosphatase that modulates immune response (Stewart et al., 1982). It consists of a catalytic and a regulatory subunit that form a functional heterodimer. Three different genes, namely, PPP3CA, PPP3CB and PPP3CC, encode the regulatory subunit and two genes, PPP3R1 and PPP3R2 encode the regulatory subunit (Rusnak and Mertz, 2000). The expression of the subunits is different in different tissues as well as in different physiological and pathological states, with the brain possessing a very high expression of all subunits (Klee et al., 1979). At low calcium concentrations, the active site of the catalytic subunit of calcineurin is blocked by an autoinhibitory domain; this inhibition is relieved when an increase in calcium concentration occurs (Hashimoto et al., 1990). In its active form, calcineurin dephosphorylates its target proteins, which are mainly transcription factors, ion channels and receptors. The best-investigated calcineurin target is the nuclear factor of activated T cells (NFaT) but also other transcription factors like cyclic AMP response element (CREB), myocyte-specific enhancer factor 2 (MEF2) transcription factor EB (TFEB) or forkhead transcription factors (FOXOs) are well-known calcineurin targets (Rao et al., 1997; Tao et al., 1998; Medina et al., 2015; Sakuma and Yamaguchi, 2010; Ni et al., 2006). Additionally, calcineurin can regulate for example NMDA receptor activity or L-type calcium channels (Tong et al., 1995; Schuhmann et al., 1997). Upon dephosphorylation, the activity of the targets can be either stimulated or blocked. For example, NFaT translocates to the nucleus and promotes expression of target genes whereas dephosphorylation of CREB reduces its activity (Tao et al., 1998; Kar et al., 2016).

Calcineurin signaling is tightly regulated temporally and spatially as previously summarized by Ulengin-Talkish and Cyert (2023). For example, on subcellular level signaling occurs in micro domains where calcineurin, its substrates and Ca^2+^ co-localize. For example, in proximity to the plasma membrane, in endocytotic compartments, in mitochondria or near centrosomes and cilia. The functions of calcineurin in different organ systems comprise a broad range of physiologic aspects including regulation of IL-2 expression in activated T cells and thereby modulating T cell function and differentiation (Fruman et al., 1992). In the nervous system, calcineurin regulates synaptic plasticity (Huang et al., 2022). In the gut, it modulates pepsinogen release or gastric acid secretion (Itoh et al., 2002; Raufman et al., 1997). Moreover, in the bone it plays an important role in osteoblast differentiation (Huynh and Wan, 2018).

Another prominent function for calcineurin has been identified in the cardiovascular system. Molkentin et al. showed that calcineurin promotes the development of cardiac hypertrophy (Molkentin et al., 1998). The vascular remodeling effects in response to stimulation with vasoactive agents like phenylephrine, endothelin or angiotensin II (angII) are calcineurin dependent (Li et al., 2017; Pang and Sun, 2009; Esteban et al., 2011). Furthermore, calcineurin regulates inflammatory processes in the vasculature. For example, some studies show that increased expression of VCAM1 (vascular cell adhesion molecule 1) and MCP1 (monocyte chemoattractant protein 1) is modulated by calcineurin/NFaT signaling (Orr et al., 2009; Satonaka et al., 2004). In renal vasculature, calcineurin stimulates extracellular matrix secretion or can influence vascular tone (Frank et al., 2006; Gooch et al., 2004). Collectively, these findings suggest that calcineurin may contribute to the development of cardiovascular diseases. In this context, peptide hormones like angII and endothelin are able to promote calcineurin activation. AngII is one effector of the renin angiotensin aldosterone system (RAS) known for its role in regulating vascular tone and sodium reabsorption.

Much of the knowledge about calcineurin function originates from studies with inhibitors that block the phosphatase activity of calcineurin, with NFaT activity being a frequently used surrogate for calcineurin activity. Several exogenous calcineurin inhibitors exist that are used as immunosuppressant (Azzi et al., 2013). Most of these inhibitors like Cyclosporine A and Voclosporine or Tacrolimus (FK-506) and Pimecrolimus are forming a complex with intracellular immunophilins like cyclophilin and FK-binding proteins that can contribute to many cellular processes (Siekierka and Sigal, 1992). These complexes inhibit the phosphatase activity of calcineurin. Altogether, around 100 calcineurin substrates are discovered until now. Among these are NFaT, cytoskeleton-associated molecules as Tau and MAP2 or cell cycle/apoptosis related targets like Cdc20 and Drp1 (Cribbs and Strack, 2007; Goto et al., 1985; Heim et al., 2018). All of these calcineurin targets are affected and no longer dephosphorylated when applying calcineurin inhibitors. This fact and the interaction of the calcineurin inhibitors with immunophilins lead to many pathophysiological calcineurin-dependent effects and effects that are unrelated to calcineurin activity inhibition. One pathological effect of calcineurin inhibitor use is the development of hypertension (Hoorn et al., 2012). Here, inhibition of calcineurin, for example, stimulates sympathetic activity, increases sodium reabsorption or, on the long term stimulates VSMC hypertrophy and extracellular matrix secretion.

## 2 Pathological effects of calcineurin activators in vascular smooth muscle cells

Smooth muscle cells maintain vascular tone in medium and small arterial blood vessels. Their ability to contract and relax allows the maintenance of blood pressure. Dysregulation of their function contributes to the development of many clinical manifestations like cardiovascular diseases (CVD) as summarized in (Nolze et al., 2023). Especially the activation of calcineurin by angII in VSMCs has been studied extensively in animal models or in cell culture experiments (Min et al., 2009; Nolze et al., 2021; Suzuki et al., 2002). Furthermore, phenylephrine, endothelin-1 or shear stress can play a role in calcineurin activation in VSMCs (Li et al., 2017; Pang and Sun, 2009). Overall, calcineurin activation in VSMC promotes CVDs by influencing differentiation, senescence, inflammatory processes as well as migration and proliferation. The first indications that calcineurin contributes to pathophysiological effects in VSMCs came from Suzuki et al. (2002). The authors show that angII activates calcineurin/NFaT-mediated gene expression and that these changes contribute to differentiation and hyperplasia. They describe that angII induces DNA binding activity of NFaT through calcineurin activation which then leads to increased gene expression of nonmuscle-type myosin heavy chain B (NMMHCB) in rat aortic VSMCs, which is known to influence phenotypic modulation of VSMCs. The proof was given by the administration of valsartan, an AT_1_-receptor antagonist, which attenuated the angII-effect. In addition, vascular senescence is influenced by angII/calcineurin. Treatment of mice with angII promoted p53 and p21 expression, which led to rise in senescent VSMCs in aortae of respective mice. Treatment of the mice with ATRAP, the AT_1_ receptor associated protein, abolished the angII effect (Min et al., 2009).

A participation of angII and calcineurin in the development of vascular inflammation processes is also documented. Satonaka et al. showed that MCP-1 expression is angII- and calcineurin-dependently regulated in VSMCs (Satonaka et al., 2004). Not only transfection with constitutively active calcineurin stimulated MCP-1 expression but also the incubation with angII itself. This effect was completely blocked after calcineurin inhibition. These findings were confirmed in a mouse model. In wire-injured femoral arteries, calcineurin inhibition suppressed neointima formation and macrophage infiltration (Satonaka et al., 2004), suggesting that calcineurin mediates vascular inflammation.

A study from Nieves-Cintron showed the involvement of calcineurin in the development of angII-mediated hypertension in myocytes of mesenteric and cerebral arteries. Increased PKCα-induced Ca^2+^ sparklets occurred after angII-administration and led to increased Ca^2+^ entry in cell culture experiments, and to higher arterial wall [Ca^2+^]_i_ and increased vascular tone in mouse experiments. This process was calcineurin/NFaT dependent (Nieves-Cintron et al., 2008).

Another study provided hints, that angII and calcineurin increase VSMC migration and thereby contribute to vessel remodeling via RCAN1 (regulator of calcineurin) after vascular injury in a mouse model (Esteban et al., 2011). AngII treatment increased motility of isolated VSMCs in a transwell migration assay by activation of calcineurin/NFaT signaling and RCAN1 expression. Additionally, the elevated migration contributed to arterial wall remodeling, namely intima thickening with a reduction in lumen area which contributes to the development of hypertension.

In our working group, we also detected the involvement of calcineurin in VSMC migration and proliferation after angII treatment together with increased Serpine1 expression. Long-term angII infusion in calcineurin WT mice led to aortic media thickening followed by hypertension. On a molecular level, angII stimulated the activity of the catalytic beta subunit of calcineurin followed by activation of the EGFR signaling pathway and consecutive upregulation of Tgfb1 and connective tissue growth factor (Ctgf) signaling. In calcineurin KO mice, the angII-driven effects were gone (Nolze et al., 2021). A more recent study confirmed the requirement of calcineurin for angII-induced hypertension and identified calcineurin-mediated changes in gene expression, independent of its phosphatase activity, as underlying molecular mechanism (Yunes-Leites et al., 2025). The authors showed that approximately 90% of the genes regulated upon angII treatment in the aorta/vascular smooth muscle cells required calcineurin expression. This indicates the importance of calcineurin in overall vascular smooth muscle cell function.

Calcineurin activation by phenylephrine (PE) can also lead to functional alterations in VSMCs. Li et al. showed in rat aortic VSMCs that PE induces calcineurin activation followed by increased cell proliferation (Li and Sun, 2005). Findings by Pang and Yaghi support these results and demonstrate that PE leads to calcineurin-dependent translocation of NFAT into the nucleus of respective cells (Pang and Sun, 2009; Yaghi and Sims, 2005). A further study showed a participation of phenylephrine-induced calcineurin activation in vascular stiffness. There, PE stimulated COX-2 expression calcineurin-dependently in VSMCs and resulted in a higher vascular tone and stiffness of aorta and mesenteric arteries (Garcia-Redondo et al., 2018).

In addition, endothelin-1 (ET-1) can promote calcineurin activity. However, studies with ET-1 focus on its role in pulmonary arterial smooth muscle cells. These reports implicate a stimulation of VSMC proliferation in a calcineurin-dependent manner (Li et al., 2017; Lu et al., 2013). However, altered vascular stiffness was also observed after calcineurin activation by ET-1. In VSMCs, ET-1 can induce Ca^2+^ influx via acid-sensing ion channel-1 and promote pulmonary arterial hypertension involving calcineurin and NFaT (Gonzalez et al., 2016).

Not only vasoactive substances but also mechanical stimuli can activate calcineurin and influence VSMC behavior. A study from Zhang et al. demonstrated a shear stress induced calcineurin activation by Ca^2+^ permeable Piezo1 channels. Higher Piezo1 activity contributes to a calcineurin-dependent increase of VSMC migration and proliferation via YAP/TAZ signaling pathways, which supported the development of neointima hyperplasia (Zhang et al., 2025).

Platelet-derived growth factor (PDGF-BB) induces calcineurin and subsequent NFaT activation that consequently lead to an upregulation of protein C receptor (PROCR) in VSMCs. Vascular injury induced the same outcome. Overall, the authors identified PROCR as NFaT target gene in VSMCs and proposed its participation in smooth muscle cell phenotypic modulation (Lee et al., 2011).

Summarized, AngII activates calcineurin-dependent signaling cascades that stimulate the expression of genes responsible for VSMC differentiation, migration and proliferation and favor inflammatory processes. Phenylephrine and ET-1 promote VSMC proliferation and contraction in a calcineurin-dependent mechanism. Consequently, these pathophysiological alterations in VSMC function contribute to the development of CVD especially of hypertension.

Another way of investigating calcineurin effects in VSMCs is with the help of calcineurin inhibitors. Therefore, we will focus in the next chapter on the pathological effects of calcineurin inhibitors on VSMC function.

## 3 Dysregulation of vascular smooth muscle cell function by calcineurin inhibitors

Calcineurin inhibitors like Cyclosporine A and Tacrolimus are important tools to reduce allograft rejection after organ transplantation and are also used for treatment of certain autoimmune diseases. However, the development of serious side effects like hypertension and nephrotoxicity after their application is still not completely understood (Xue et al., 2014). Initially, both drugs decrease the production of IL-2, a pro-inflammatory cytokine by calcineurin inhibition. Additionally they reduce the activation of lymphocytes (Bra et al., 2010). Unexpectedly, these inhibitors only sometimes have opposite effects when compared to that of the activators. However, very often calcineurin inhibitors show controversial effects mimicking that of activators.

Especially for the development of hypertension, an increased contractility in VSMCs after calcineurin inhibitor application was identified. This can lead to elevated vasoconstriction and in the end to hypertension. Grzesk et al. provided as explanation, that CsA directly stimulate PKC activity in smooth muscle cells, which increases the calcium concentration influx from extracellular stores into the cytoplasm of the respective cells (Grzesk et al., 2012). In a more recent study, the same authors compared the effect of CsA and Tacrolimus on smooth muscle cell contractility and showed that Tacrolimus did not increase calcium influx significantly (Grzesk et al., 2016). Another group could show that Tacrolimus inhibits the big-conductance Ca^2+^ activated K^+^ channels. These channels are important for membrane hyperpolarization, and inhibition of voltage dependent Ca^2+^ channels. In turn, extracellular Ca^2+^ influx is reduced and the smooth muscle cell can relax. The inhibition of these channels by Tacrolimus reduces smooth muscle cell relaxation and thereby increases vascular tone that favors the development of hypertension (Tang et al., 2021). Similarly, the effect of tacrolimus on voltage gated K^+^ (K_v_) channels was investigated. The authors could show that Tacrolimus inhibits the K^+^ current through the K_v_ channels in a concentration dependent manner. Additionally the effect was present after previous calcineurin inhibition with CsA, indicating that the K_v_ channel inhibition is a calcineurin-independent effect of Tacrolimus (Li et al., 2019). In addition to direct smooth muscle effects, inhibition of endothelial NO release can cause pathological outcomes and influence contractility of vascular smooth muscle cells. Endothelial nitric oxide synthase shows also a calcineurin inhibitor sensitivity, as different studies demonstrated (Can et al., 2007; Lopez-Ongil et al., 1996). A reduced availability of NO in smooth muscle cells and thereby increased vascular tone is another possible feature of calcineurin inhibitor associated pathophysiology. Another possible explanation for increased vascular tone comes from a more recent study (Wang et al., 2022). A pre-incubation of mouse aortic VSMCs with Tacrolimus activated the RhoA/ROCK/MYPT-1 pathway, which is directly involved in the development of hypertension, and increased vascular tone (Wang et al., 2022; Kawarazaki and Fujita, 2021). Additionally, Tacrolimus induced ROS production and thereby potentiated RhoA/ROCK/MYPT-1 signaling. Nevertheless, also altered proliferation of VSMCs is reported upon calcineurin inhibitor usage. The first results on this issue were controversial, as the smooth muscle cell proliferation after calcineurin inhibitor usage decreased or increased (Barnhart et al., 1987; Jonasson et al., 1988). For Cyclosporine A, a study by Leszczynski et al. suggested a time- and dose-dependent effect on rat smooth muscle cell proliferation (Leszczynski et al., 1993). A dose of around 10‑^7^ M induced VSMC proliferation. This concentration is comparable to those that are present in patients treated over a long period with CsA. In addition, Tacrolimus is also able to promote VSMC proliferation as a study from Giordano showed (Giordano et al., 2008). Here, Tacrolimus stimulated collagen type I production, Erk and Akt signaling. Especially TGFbeta signaling was enhanced and increased proliferation.

Despite the above-described controversies, calcineurin inhibitors can reverse certain inflammatory, proliferative and migratory processes in the vascular wall. Initially, an anti-inflammatory role upon CsA and tacrolimus application in rat VSMC was reported. Akita et al. showed in the early 90s that CsA, and to a lesser extend Tacrolimus, lead to reduced induction/expression of NO synthase (NOS2) followed by reduced NO production after LPS stimulation in smooth muscle cells (Akita et al., 1994). Additional findings demonstrated that CsA reduces inducible nitric oxide synthase mRNA levels without affecting mRNA stability upon cytokine treatment. Again, Tacrolimus had a weaker effect (Marumo et al., 1995). Later a study suggested a transcriptional mechanism for CsA but a post-transcriptional effect for Tacrolimus in NOS2 regulation (Dusting et al., 1999). Another group reported about the inhibition of smooth muscle cell proliferation by Tacrolimus in 2006. Upon Tacrolimus treatment with a concentration of 10^–5^ M, the expression of cell cycle inhibitor p27 in PDGF-stimulated VSMCs increased and led to a cell cycle arrest (Matter et al., 2006). Furthermore, Garvey et al. found an influence of CsA on phenotypic modulation of smooth muscle cells with reduced proliferation. Cyclosporine A treatment upregulated the expression of transcription factor KLF-4 (Krüppel-like factor-4) which inhibits proliferation but promotes migration. Additionally, KLF-4 downregulated alpha-smooth muscle actin and smoothelin (Garvey et al., 2010). By this, phenotypic switch of smooth muscle cells alters. Several reports describe an inhibitory effect of Tacrolimus on VSMC proliferation and migration. Ma et al. found a Tacrolimus-mediated effect in PDGF-BB treated rat VSMCs in a concentration-dependent manner (Ma et al., 2017). Concentrations of 2–5 μg/mL Tacrolimus together with 10 ng/mL PDGF-BB could inhibit proliferation and migration of VSMCs without being cytotoxic. Other data show, that, for example, tacrolimus eluting stents can prevent restenosis after vascular injury (Mok et al., 2025; Park et al., 2024). These stents continuously release the respective drug to reduce VSMC proliferation. A more recent study evaluated the efficacy of tacrolimus eluting stents and demonstrated an inhibitory effect on VSMC proliferation and migration (Park et al., 2024). Another calcineurin inhibitor used in clinics is Pimecrolimus. This compound differs just in one amino acid compared to Cyclosporine A and shows a more specific binding to calcineurin. It has anti-inflammatory and immunomodulatory effects and got approval for treatment of skin diseases (Luger et al., 2001). Hussner et al. found that Pimecrolimus inhibited proliferation in human VSMCs *via* upregulation of IFN-β (Hussner et al., 2016).

Despite the successful application of calcineurin inhibitors in the clinics, unwanted cardiovascular side effects accompany the usage of Cyclosporine A and Tacrolimus. That dilemma led to the development of a class of new calcineurin inhibitors that circumvent the full inhibition of catalytic calcineurin activity. This class of therapeutics, called VIVIT, mimics the NFaT recognition motif (PxIxIT) and blocks the NFaT binding site in calcineurin (Aramburu et al., 1999; Yu et al., 2007). By this, VIVIT prevents NFaT dephosphorylation without affecting calcineurin phosphatase activity on other targets. In different studies, the effectiveness of VIVIT was tested and led to the assumption that this new class of inhibitors reduced pathophysiologic relevant effects dramatically (Kuriyama et al., 2006; Zhang et al., 2013). In vascular smooth muscle cells, the application of VIVIT is not well described. Several studies show, that VIVIT efficiently can block NFaT signaling, for example, with respect to cell cycle progression of smooth muscle cells to avoid restenosis after vascular injury or in formation of intracranial aneurysms by a phenotypic switch of respective smooth muscle cells (Karpurapu et al., 2008; Sun et al., 2023). We just can speculate, that the previously discussed issues after application of the common calcineurin inhibitors CsA and Tacrolimus are less pronounced or in the best case are completely absent by usage of VIVIT derived peptides.

## 4 Conclusions and perspectives

**FIGURE 1:**
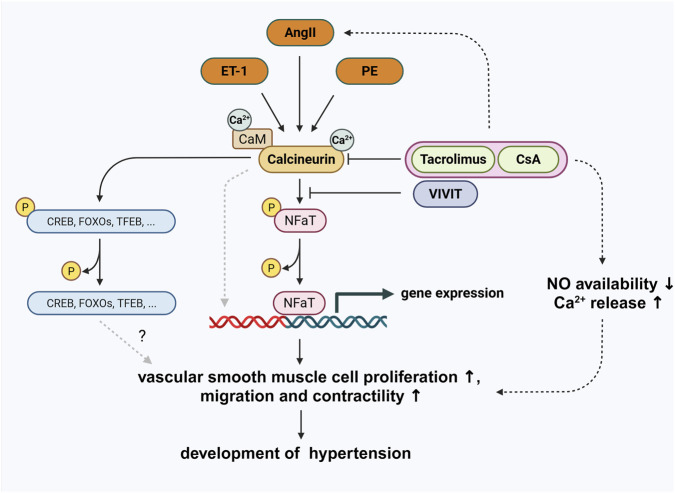
Overview of the pathological effects of calcineurin activators and calcineurin inhibitors on vascular smooth muscle cell function. Dashed black lines indicate calcineurin independent inhibitor effects with steps in between that are not displayed. Dashed grey lines indicate calcineurin-dependent effects. Only a fraction of intracellular signaling pathways affected by calcineurin is shown. Created with BioRender; license for Alexander Nolze.

A pathological activation of the serine/threonine phosphatase calcineurin by angII, phenylephrine, endothelin-1 or shear stress can alter VSMC behavior and can lead to severe consequences like increased vascular tone, inflammation, migration and proliferation and remodeling processes. Interestingly, calcineurin inhibitors like Cyclosporine A or Tacrolimus only sometimes inhibit these effects but instead can lead to similar pathological outcomes in VSMCs with similar clinical consequences as calcineurin activators (Figure 1). The differentiation between calcineurin-dependent and -independent effects of calcineurin inhibitors on VSMC function is not easy and often it seems unclear if the described effects come from calcineurin inhibition itself or from calcineurin inhibitor effects on other unrelated targets.

Especially proliferation, migration and contractility of the cells is increased after drug application, which suggest calcineurin-independent effects. A possible explanation for these common features is that calcineurin inhibitors, just like calcineurin itself, may stimulate the renin angiotensin system. The systemic RAS encompasses many cell types and components and originates from the kidney. However, a local RAS exists in the vascular wall and VSMCs can express several components. For example, the cells can produce angII locally. One model assumes a stimulation of the RAS by calcineurin inhibitors. In non-VSMCs, the effects are driven for example by a direct activation of renin production (Prokai et al., 2016). Interestingly, a clinical study showed that RAS inhibition and avoidance of high doses of calcineurin inhibitors could prevent interstitial fibrosis in kidney transplant patients (Sayin et al., 2017). Furthermore, in a rat model, RAS inhibition, especially angII receptor inhibition, restored COX-2 expression after calcineurin inhibitor application (Hu et al., 2021). However, this so-called calcineurin-inhibitor induced RAS hyperactivity is not well described in VSMCs. Avdonin et al. showed a stimulatory effect of Cyclosporine A on AT_1_ receptor abundance in human aortic vascular smooth muscle cells but this effect was calcineurin-independent (Avdonin et al., 1999). Another explanation for the described calcineurin inhibitor effects is that they only inhibit the enzymatic activity of calcineurin but do not affect the non-enzymatic modulation of gene expression. For example, angII-induced hypertension is calcineurin-dependent in mice, but CsA treatment did not abolish this effect (Yunes-Leites et al., 2025). A subset of angII-regulated genes was identified that required calcineurin expression but not its phosphatase activity. This could also explain the same effects after calcineurin activation and calcineurin inhibition. Another reason for the observed inhibitor effects is the binding to the immunophilins cyclophilin and FK-binding protein, which are then no longer available for other interactions. Immunophilins are important endogenous peptidyl-prolyl-cis/trans isomerases and are necessary for cellular processes. Negative effects due to the binding to immunophilins are for example, a disruption of mitochondrial transition pore formation (Tanveer et al., 1996). Furthermore, for CsA, off target effects on apoptosis related proteins caspase 3 or p38 MAP kinase 14 are reported (Hu et al., 2014).

A more targeted approach with the use of VIVIT, a peptide blocking NFAT binding site in calcineurin seemed promising, but has not been approved for clinical use due to challenges in drug development. Another possibility for inhibition of calcineurin signaling with less pathological effects could be a isoform specific inhibition of NFaT by blocking specific binding regions in calcineurin for the interaction with NFaT1-4 (Kitamura and Kaminuma, 2021; Kitamura et al., 2020; Martinez-Martinez et al., 2006). For example, NFAT3 shows a high expression in VSMCs and seems to be a promising target for an isoform specific inhibition. This could avoid the unwanted pathophysiological effects in the respective cell type.

Overall, separate effects induced by calcineurin activation and inhibition in VSMCs and other cell types and their interplay will influence vascular contraction and vascular remodeling and thereby affect the clinical outcome.

Nevertheless, to overcome the dysfunction of VSMCs after calcineurin activation and/or calcineurin inhibition and their contribution to the development of hypertension, a well-considered medication is necessary. To prevent this, a more specific inhibition of calcineurin function would be required but most of these isoform specific drugs have not reached clinical application yet.
